# Case Report: Longitudinal follow-up and testicular sperm extraction in a patient with a pathogenic *NR5A1* (SF-1) frameshift variant: p.(Phe70Serfs*5)

**DOI:** 10.3389/fendo.2023.1171822

**Published:** 2023-06-20

**Authors:** Jordan Teoli, Delphine Mallet, Lucie Renault, Claire-Lise Gay, Elsa Labrune, Patricia Bretones, Sandrine Giscard D’Estaing, Béatrice Cuzin, Frédérique Dijoud, Florence Roucher-Boulez, Ingrid Plotton

**Affiliations:** ^1^ Service de Biochimie et Biologie Moléculaire, Unité Médicale de Biologie Endocrinienne, Centre de Biologie et Pathologie Est, Hospices Civils de Lyon, Bron, France; ^2^ Département des sciences biomédicales B, Institut des sciences pharmaceutiques et biologiques, Université Claude Bernard Lyon 1, Lyon, France; ^3^ Institut Cellule Souche et Cerveau (SBRI), Unité de Institut national de la recherche médicale (INSERM) 1208, Centre de Recherche INSERM, Bron, France; ^4^ Centre de Référence Maladies Rares du Développement Génital: du Fœtus à l’Adulte, Filière Maladies Rares Endocriniennes, Bron, France; ^5^ Service de médecine de la reproduction, Hôpital Femme-Mère-Enfant, Hospices Civils de Lyon, Bron, France; ^6^ Service d’endocrinologie pédiatrique, Institut Saint-Pierre, Palavas-Les-Flots, France; ^7^ Faculté de médecine, Université Claude Bernard Lyon 1, Lyon, France; ^8^ Service d’endocrinologie pédiatrique, Hôpital Femme-Mère-Enfant, Hospices Civils de Lyon, Bron, France; ^9^ Chirurgie Urologique, Centre Lyonnais d’Urologie Bellecour, Lyon, France; ^10^ Service d’Anatomie Pathologique, Centre de Biologie et de Pathologie Est, Hospices Civils de Lyon, Bron, France; ^11^ Institut Génétique, Reproduction & Développement (iGReD), Centre national de la recherche scientifique (CNRS), INSERM, Université Clermont Auvergne, Clermont–Ferrand, France

**Keywords:** testicular sperm extraction, gonadal dysgenesis, spermatogenesis, male infertility, congenital, disorder of sex development, hypospadias, azoospermia

## Abstract

**Background:**

Steroidogenic factor 1 (SF-1), encoded by the nuclear receptor subfamily 5 group A member 1 (*NR5A1*) gene, is a transcriptional factor crucial for adrenal and gonadal organogenesis. Pathogenic variants of *NR5A1* are responsible for a wide spectrum of phenotypes with autosomal dominant inheritance including disorders of sex development and oligospermia–azoospermia in 46,XY adults. Preservation of fertility remains challenging in these patients.

**Objective:**

The aim was to offer fertility preservation at the end of puberty in an *NR5A1* mutated patient.

**Case report:**

The patient was born of non-consanguineous parents, with a disorder of sex development, a small genital bud, perineal hypospadias, and gonads in the left labioscrotal fold and the right inguinal region. Neither uterus nor vagina was detected. The karyotype was 46,XY. Anti-Müllerian hormone (AMH) and testosterone levels were low, indicating testicular dysgenesis. The child was raised as a boy. At 9 years old, he presented with precocious puberty treated by triptorelin. At puberty, follicle-stimulating hormone (FSH), luteinising hormone (LH), and testosterone levels increased, whereas AMH, inhibin B, and testicular volume were low, suggesting an impaired Sertoli cell function and a partially preserved Leydig cell function. A genetic study performed at almost 15 years old identified the new frameshift variant NM_004959.5: c.207del p.(Phe70Serfs*5) at a heterozygous state. He was thus addressed for fertility preservation. No sperm cells could be retrieved from three semen collections between the ages of 16 years 4 months and 16 years 10 months. A conventional bilateral testicular biopsy and testicular sperm extraction were performed at 17 years 10 months of age, but no sperm cells were found. Histological analysis revealed an aspect of mosaicism with seminiferous tubules that were either atrophic, with Sertoli cells only, or presenting an arrest of spermatogenesis at the spermatocyte stage.

**Conclusion:**

We report a case with a new *NR5A1* variant. The fertility preservation protocol proposed at the end of puberty did not allow any sperm retrieval for future parenthood.

## 1 Introduction

Steroidogenic factor 1 (SF-1) is a transcription factor crucial for adrenal and testis organogenesis as well as steroidogenesis regulation with a dose-dependent effect (1–3).

SF-1 protein is encoded by the nuclear receptor subfamily 5 group A member 1 (*NR5A1*) gene located in chromosome 9 and composed of seven exons. SF-1 protein is characterised by a DNA-binding domain (DBD) in the amino-terminal region and by a ligand-binding domain (LBD) in the carboxy-terminal region separated by a hinge region, which can host post-translational changes (2, 4, 5).

To date, more than 180 putative pathogenic variants have been reported in *NR5A1* coding regions and splice sites, spanning the whole gene and including missense variants (58% of variants), frameshift variants (18.6%), non-sense variants (12.3%), and splice variants (3.3%) in a heterozygous and isolated state in almost all cases. Variants were *de novo* in almost half of the cases and autosomal dominant inheritance in the others (4).

Pathogenic variants in *NR5A1* are responsible for almost 20% of 46,XY differences or disorders of sex development (DSDs) (4). 46,XY DSD related to mutated *NR5A1* is characterised by a wide spectrum of phenotypes, from male to female external genitalia and including partial or complete dysgenesis, genital ambiguity, micropenis, hypospadias, cryptorchidism, and asplenia with no clear genotype–phenotype correlation. Adrenal insufficiency is rarely associated with the picture (4, 6).

Severe oligospermia or azoospermia can be found in 46,XY patients carrying *NR5A1* pathogenic variants; sometimes, these are the only symptoms (7, 8). Fertility care in 46,XY patients presenting azoospermia and carrying *NR5A1* pathogenic variants has rarely been studied. Among these patients, only four cases who underwent testicular sperm extraction (TESE) have been reported, with inconsistent results (**Table 1**), underlining that preservation of fertility in such cases is challenging.

**Table 1:** Fertility preservation in mutated *NR5A1* 46,XY men presenting azoospermia.

Patient	*NR5A1* variant (NM_004959.5, GRCh37/hg19)	Clinical description at birth	Hormonal description (closest to TESE)	Gonadal description	TESE procedure and results (age)	Reference
**1**	c.118A>C p.(Thr40Pro) Heterozygous	Ambiguous genitalia, palpable gonads in labioscrotal folds, no Müllerian remnant at birth	FSH ↗ LH ↗ Testosterone subN Inhibin B ↘	Hypoplastic testis, neonatal testicular appearance on histology at birth Low TV (L and R: 4-6 ml) at 15.8 y-o	TESE: no sperm cells (18 y-o)	(9)
**2**	c.39C>A p.(Cys13*) Heterozygous	Bilateral cryptorchidism treated by orchidopexy	FSH ↗ LH N Testosterone N	Low TV (L: 6.6 ml, R: 10.1 ml) at adult age	Micro-TESE after 3 months of vitamin E and clomiphene citrate: few sperm cells, but the number was not specified (20 y-o)	(10)
**3**	c.730A>G p.(Ile244Val) Heterozygous	Not available	FSH N LH N Testosterone N	Normal TV (L: 13.4 ml, R: 14.1 ml) at adult age	Micro-TESE after 3 months of vitamin E and clomiphene citrate: few sperm cells, but the number was not specified (35 y-o)	(10)
**4**	c.244+1G>A Heterozygous	Not available	FSH ↗ LH ↗ Testosterone subN	Low TV (L: 4.1 ml, R: 5.4 ml), normal spermatogenic function on histology at adult age	Micro-TESE: sufficient sperm cells for ICSI (31 y-o)	(10)

FSH, follicle-stimulating hormone; ICSI, intracytoplasmic sperm injection; L, left; LH, luteinising hormone; N, normal; R, right; subN, subnormal; TESE, testicular sperm extraction; TV, testicular volume; y-o, years old; ↘ or ↗, decreased or increased, respectively.

Here, we report a longitudinal follow-up from birth to adulthood of a patient carrying a novel frameshift variant of *NR5A1.* We focused our follow-up on the physical and hormonal evaluations particularly during the puberty period, and also on testicular histology and semen collections.

## 2 Case report

The patient was born to non-consanguineous parents at 37 weeks of amenorrhea with a tetralogy of Fallot and a DSD. Longitudinal morphological and laboratory data are summarised in **Table 2**.

**Table 2:** Birth and follow-up morphological and laboratory data in a patient with a pathogenic *NR5A1* (SF-1) frameshift variant.

Age	Ongoing treatment	Height, weight, BMI	Penis length (mm)	Testicular volume	FSH (IU/L)	LH (IU/L)	Total testosterone (nmol/L)	Inhibin B (ng/L)	AMH (pmol/L)	Bioavailable testosterone (nmol/L)	Other examinations
**H12**	/	46 cm, 2.320 kg	6	9 × 6 mm (L) Not palpated (R)	/	/	0.90 ↘	/	64.0 ↘	/	/
**D2**	/	/	/	/		/	<0.10 (N)	/	/	/	/
**D14**	/	/	/	/	9.5 ↗	9.2 ↗	3.02 (subN)	/	/	/	/
**D27**	After 7 injections of hCG	/	16	/	/	/	10.85 (with parallel increase in dihydrotestosterone) (N)	/	92.5 ↘	/	/
**1y**	After 11 injections of testosterone	/	32	/	/	/	/	/	/	/	Tanner P2
**9y**	/	/	/	35 × 18 mm (L and R)	/	/	9.00 (↗ for age)	<30 ↘	18.7 (↘)	/	Bone age: 11y6m
**9y10m**	Triptorelin	BMI at 97e percentile	45	/	/	/	/	/	/	/	/
**11y4m**	Triptorelin		/	/	/	/	/	/	/	/	Bone age: 13y
**12y7m**	/		50	33 × 16 mm (L) 32 × 15 mm (R)	22.7 ↗	10.6 ↗	/	6 ↘	1.3 ↘	/	Tanner P4
**13y6m**	/	160.5 cm, 60.5 kg BMI: 23.5 (>97e percentile)	55	25 × 20 mm (L) 28 × 20 mm (R)	36 ↗	18.5 ↗	13.63 N	/	/	/	Tanner P4 Bone age: 13y6m
**14y10m**	Testosterone	163 cm	55-60	8 ml (L) 6 ml (R)	/	/	/	< 5 ↘	0.8 ↘	/	Bone age: 15y–16y
**15y1m**	/	/	/	/	29.5 ↗	25.0 ↗	9.46 ↘	/	/	/	/
**16y4m**	/	/	/	6 ml (L) 6 ml (R)	33.2 ↗	19.8 ↗	6.50 ↘	9.0 ↘	2.6 ↘	2.10 (subN)	SBP: 10 nmol/L
**16y8m**	/	165 cm, 65 kg BMI: 23.9 (around 90e percentile)	/	/	32.8 ↗	20.1 ↗	9.40 ↘	6.0 ↘	2.5 ↘	2.71 (subN)	SBP: 16 nmol/L
**16y10m**	/	/	/	/	29.4 ↗	19.0 ↗	13.06 N	7.0 ↘	2.5 ↘	4.38 (N)	SBP: 17 nmol/L
**20y**	/	172 cm (for a genetic target at 174 cm), 82 kg BMI = 27.7	/	/	/	/	/	/	/	/	/

AMH, anti-Müllerian hormone; D, day; FSH, follicle-stimulating hormone; H, hour; hCG, human chorionic gonadotropin; L, left; LH, luteinising hormone; m, month; N, normal; R, right; SBP, sex-binding protein; subN, subnormal; y, year; BMI, body mass index; ↘ or ↗, decreased or increased, respectively, according to reference range for age and sex when it exists or personal interpretation (in parentheses) when it does not.

Plasma FSH and LH were assessed by radioimmunoassay (newborn data) or by an automated chemiluminescence immunometric assay on Architect i2000SR (Abbott, Chicago, IL, USA). Reference ranges for FSH: 0.05 to 1 IU/L in prepubescent boys between 10 and 30 months, 1.1 to 7.2 IU/L in men with normal testicular function. Reference ranges for LH: 0.02 to 0.80 IU/L in prepubescent boys between 10 and 30 months, 1.3 to 5.8 IU/L in men with normal testicular function.

Plasma total testosterone was assessed by in-house radioimmunoassay after solvent extraction and chromatography or by in-house liquid chromatography coupled with tandem mass spectrometry after extraction. Reference ranges for boys: 9.36 ± 5.31 nmol/L at D1, 0.97 ± 0.38 nmol/L at D5, 5.37 ± 2.64 nmol/L between D11 and D15, 8.68 ± 2.77 nmol/L between 1 and 3 months, 0.28 ± 0.01 nmol/L (mean ± standard deviation) in prepubescent boys between 1 and 10 years old, 10.40 to 26.00 nmol/L in young men.

Serum inhibin B was assessed by enzyme immunoassay using the Inhibin B Gen II ELISA kit (Beckman Coulter, Brea, CA, USA). Reference range: 35 to 167 ng/L in boys between 6 and 10 years old, 74 to 470 ng/L in boys between 12 and 17 years old, 92 to 316 ng/L in normozoospermic men (11, 12).

Serum AMH was assessed by enzyme immunoassay (newborn data) or by an automated electrochemiluminescence assay on Cobas e601 (Roche Diagnostics, Basel, Switzerland). Reference ranges for boys: 395 to 2,321 pmol/L between D13 and D20, 505 to 3,213 pmol/L between 2.8 and 5.1 months, 705 to 4,280 pmol/L between 8.5 and 9.8 months, 441 to 2,352 pmol/L at 4 years old, 16.4 to 90.3 pmol/L in men with normal spermatogenesis (13, 14).

Plasma bioavailable testosterone was assessed by in-house radioimmunoassay after solvent extraction and chromatography. Reference range: 2.25 to 10.70 nmol/L in men between 20 and 40 years old.

SBP was assessed by radioimmunoassay with the SHBG RIACT Cisbio Kit (Cisbio Bioassays, Codolet, France). Reference range: 17 to 45 nmol/L in men.

At birth, the patient had a small genital bud (6 mm long) with perineal hypospadias. A gonad of 9 × 6 mm was palpated in the left labioscrotal fold, and the right gonad was in the high inguinal region. The genitography did not show any uterus or vagina. Karyotype and fluorescence *in situ* hybridisation showed a normal 46,XY formula. Anti-Müllerian hormone (AMH) levels were low on the first day of life (64 pmol/L) and at the minipuberty (92.5 pmol/L). Testosterone level was also low at the 12th hour of life (0.90 nmol/L) and was stimulated to 10.85 nmol/L after a human chorionic gonadotropin (hCG) test (seven injections of 1,500 IU every 2 days). On the 14th day of life, follicle-stimulating hormone (FSH) and luteinising hormone (LH) levels were high (9.5 and 9.2 IU/L, respectively).

The patient received four injections of heptylate testosterone (two doses of 20 mg and then two doses of 25 mg, 15 days apart) and was declared male at 3 months of age. Several surgical treatments were performed, first for his hypospadias at 1 year of age, then for the undescended testis at 2 years 6 months, and finally for the correction of the penis curvature at 9 years. At 9 years, precocious puberty was suspected due to an increase in testicular volume (TV), and a gonadotropin-releasing hormone (GnRH) test confirmed a central origin. Magnetic resonance imaging of the hypothalamo-pituitary region was normal. GnRH analog treatment (triptorelin: one injection every 4 weeks and then every 3 weeks due to insufficient effectiveness) was introduced from 9 years 10 months to 11 years 4 months of age. From the age of 13 years 6 months to 14 years 10 months, testosterone enanthate (50 to 125 mg, one injection every 3 weeks) was undertaken since LH was high and to improve penis size prognosis but was interrupted because of its poor effectiveness. At 14 years old, a varicocele on the left side was observed and highlighted by testicular echography.

At 16 years 4 months of age, he was addressed for fertility preservation. TV was diminished (6 ml on both sides), and AMH and inhibin B levels were low. Three semen collections performed according to the 2010 World Health Organization criteria (15) between the ages of 16 years 4 months and 16 years 10 months retrieved no sperm cells. The varicocele on the left side was treated by embolisation at the age of 17 years 2 months. The patient was eligible for a testicular biopsy with testicular sperm extraction (conventional TESE) since no sperm cells were found in at least two sperm samples 3 months apart. TESE was practiced according to the procedure described previously (16) when the patient was 17 years 10 months old. Only one sperm cell was found on a right testis fraction, but this was insufficient for cryopreservation.

**Figure 1:**
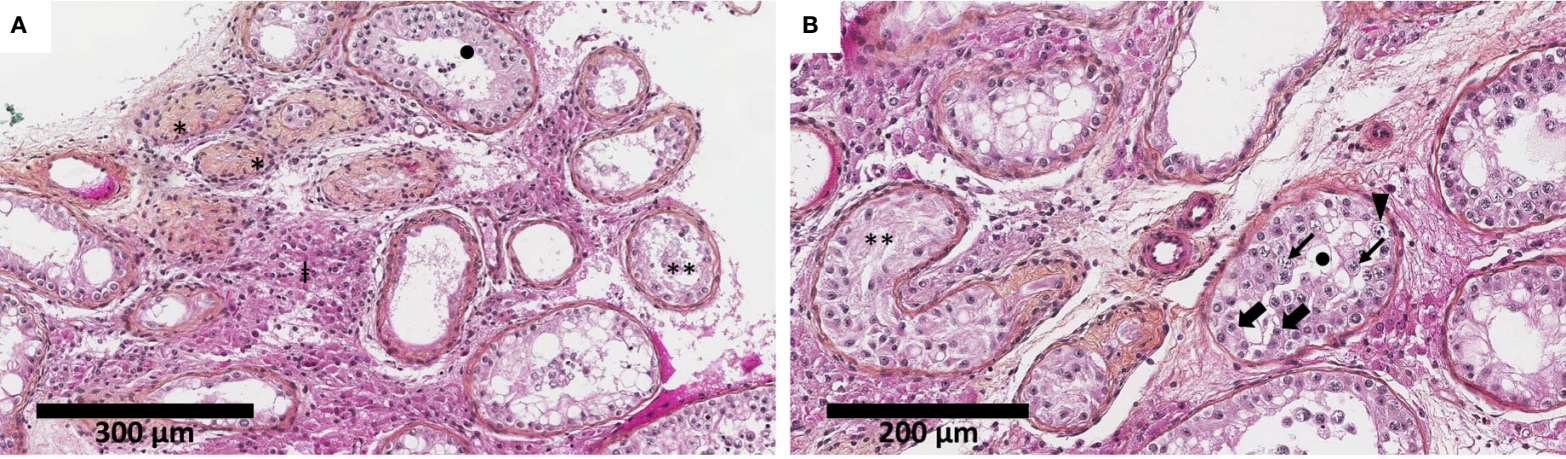
Testicular biopsy shows severely impaired spermatogenesis with an aspect of histological mosaicism. The seminiferous tubules were of overall reduced diameter (decreased by approximately 30%–50% compared to physiological adult pubertal seminiferous tubules of 150–250 µm in diameter) and lined by a thickened basal membrane. The seminiferous tubules were either atrophic (*), with Sertoli cells only (**), or presenting spermatogenesis arrest at the spermatocyte stage (•). The interstitial tissue was fibro-edematous with numerous Leydig cells (‡) (hematoxylin–eosin–saffron). Thick black arrow indicates Sertoli cells, thin black arrow indicates spermatocyte, and solid black triangle indicates spermatogonia. Biopsy fragments were fixed in alcohol, formalin, and acetic acid (AFA) and paraffin-embedded. Sections of 3 μm were stained by hematoxylin–phloxin–saffron. Slide evaluation was performed on a Leica DM2500 microscope. Two different scales: 300 µm **(A)** and 200 µm **(B)**.

The histological analysis of biopsy fragments revealed a severely impaired spermatogenesis with an aspect of histological mosaicism using Johnsen score (17): the seminiferous tubules, of overall reduced diameter and lined by a thick basal membrane, were either atrophic, with Sertoli cells only, or with spermatogenesis arrest at the spermatocyte stage. The interstitial tissue was fibro-edematous with hyperplastic Leydig cells. No signs of malignancy or dysplasia were noticed (**Figure 1**).

**Figure 2:**
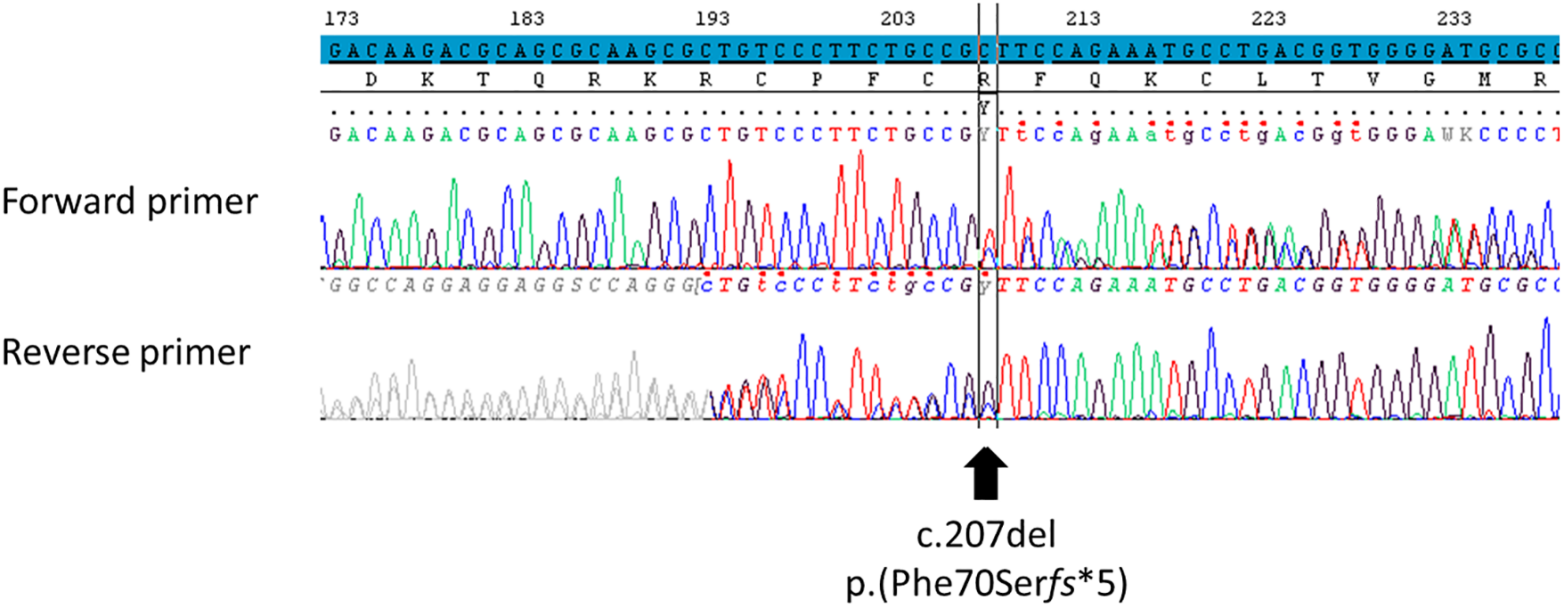
Identification of the *NR5A1* variant using Sanger sequencing. Variation was identified using reference NM_004959.5 for *NR5A1* transcript on GRCh37/hg19 human genome assembly, NP_004950.2 for SF-1 protein. The screenshot comes from SeqScape 3 software. The nucleotide reference sequence is highlighted in blue. This variant was classified as pathogenic according to the ACMG criteria (PVS1, PM1, and PM2). The polymorphism NM_004959.5: c.437G>C p.(Gly146Ala) was not found. In ACMG criteria: PM, pathogenic moderate; PVS, pathogenic very strong. “PVS1: null variant (non-sense, frameshift, canonical ±1 or 2 splice sites, initiation codon, single or multi-exon deletion) in a gene where loss of function is a known mechanism of disease.” “PM1: located in a mutational hot spot and/or critical and well-established functional domain (e.g. active site of an enzyme) without benign variation.” “PM2: absent from controls (or at extremely low frequency if recessive) in Exome Sequencing Project, 1000 Genomes or ExAC.”.

At 14 years 10 months of age, after his parents provide signed informed written consent for genetic testing, a molecular analysis of *NR5A1* gene (Sanger sequencing on DNA extracted from whole blood) revealed the unreported heterozygous frameshift variant NM_004959.5: c.207del p.(Phe70Serfs*5) (GRCh37/hg19) (**Figure 2**). His parents and his sibling were not sequenced for *NR5A1*. According to the American College of Medical Genetics and Genomics (ACMG) criteria (18), this variant is classified as pathogenic. This variant had never been reported in Gnomad_v2, ClinVar, and dbSNP databases or in any literature to date. It is in the third exon of *NR5A1* gene encoding the DBD of the SF-1 protein. Since it induces a frameshift with the manifestation of a premature stop codon, it should lead to either an inactive truncated protein (truncated DBD, absence of the hinge region and the LBD) or the absence of protein by the non-sense-mediated mRNA decay (NMD) mechanism (19). The coding regions of the androgen receptor (*AR*) gene were also studied; no pathogenic variant was found.

Otherwise, the patient had some periods of overweight during childhood and early adulthood because of a lack of physical activity and overeating. The patient never presented with an adrenal crisis and the exploration of the hypothalamic–pituitary–adrenal axis was normal (cortisol and adrenocorticotropic hormone (ACTH)).

## 3 Discussion

We describe a 46,XY male patient carrying a new *NR5A1* pathogenic variant with medical monitoring from birth to adulthood, at the beginning of which a testicular biopsy with TESE was performed.

The novel variant NM_004959.5: c.207del p.(Phe70Serfs*5) found here was classified as pathogenic according to the ACMG criteria. Certain frameshift variants inducing a premature stop codon in the same position as those observed herein have been reported to lead to a protein with the same missing parts (truncated DBD, absence of the hinge region, and the LBD) if one is produced. The variant NM_004959.5: c.18del p.(Asp6Glufs*69) was found in a 46,XY patient born with ambiguous external genitalia and raised as female. At 27 years old, she had clitorimegaly, a blind-ended vagina with no uterus, severely hypoplastic inguinal testis, primary hypogonadism with high gonadotropin levels and low baseline testosterone level non-responsive to hCG stimulation, and no adrenal dysfunction (20). Another variant, NM_004959.5: c.70del p.(His24Thrfs*51), was found in a 46,XY patient with ambiguous external genitalia at birth. The patient presented with an absence of Müllerian ducts and no palpated gonads and was initially raised as female until 18 years old. The patient had high gonadotropin levels, normal testosterone concentration, and normal adrenal function at 18 years of age, and the patient’s testes were considered dysgenetics at 19 years old (21). The last variant, NM_004959.5: c.151del p.(Glu51Argfs*24), was found in a 46,XY adolescent patient raised as female and who presented with clitorimegaly, primary amenorrhea, and inguinal testes (22). The p.(Asp6Glufs*69) and p.(His24Thrfs*51) variants were explored by functional studies that showed a reduction in the transactivation capacity of SF-1 on the promoters of some genes coding for steroidogenic enzymes. Nevertheless, Western blotting performed on transfected cells could not detect any protein, due to either NMD or the non-ability of the technique to detect small peptides (20, 21). The p.(Phe70Serfs*5) variant identified herein should have similar consequences on the transactivation capacity of SF-1 than the p.(Asp6Glufs*69) and p.(His24Thrfs*51) variants.

The clinical and hormonal data recorded in the present patient could be integrated into the wide spectrum of phenotypes of mutated *NR5A1* patients (reviewed in (4, 23)).

At the patient’s birth, AMH levels were low for a 46,XY newborn but too high for a 46,XX newborn, and total testosterone level was subnormal but increased properly after hCG stimulation in early childhood. These two parameters indicated the presence of dysgenetic testicular tissue with an impaired function more marked on Sertoli cells than on Leydig cells. However, the absence of the uterus indicated a sufficient secretion of AMH during the *in utero* life. As suggested elsewhere, foetal Sertoli cell function was thus sufficient to induce the regression of the Müllerian ducts but decreased after birth (23–25). The incompletely virilised external genitalia reported herein suggests that testosterone and dihydrotestosterone were probably insufficiently secreted during the *in utero* window of masculinisation to induce the complete development of external genitalia (26). Testosterone levels (basal or stimulated) vary greatly among 46,XY *NR5A1* mutated patients (23).

At puberty, the patient showed an insufficient increase in TV, low AMH and inhibin B levels, and elevated FSH levels that indicated a severe primary Sertoli cell injury. The normal or subnormal testosterone levels with elevated LH levels suggested a compensated primary hypofunction of Leydig cells and explained the virilisation signs observed in the patient at puberty. This description is in line with that of Mönig et al., who studied 10 *NR5A1* mutated patients during adolescence and puberty (24). Other authors also showed an impaired Sertoli cell function with a normal or subnormal Leydig cell function conserved at least until puberty (23, 25, 27).

Interestingly, the precocious increase in TV and testosterone levels herein suggested precocious puberty confirmed by a GnRH test. The occurrence of precocious puberty was surprising because SF-1 is expressed in pituitary cells in humans and is implicated in the formation of the ventromedial hypothalamic nucleus in mice (28) and because some mutated *NR5A1* patients encountered difficulties when entering puberty spontaneously (29). However, Mönig et al. reported an early pubertal development with an early increase in testosterone levels in three out of 10 cases (24).

Amazingly, the spontaneous increase in testosterone levels and virilisation at puberty contrasted with the subnormal testosterone levels and incompletely virilised external genitalia at birth reported herein and elsewhere (23, 24). As demonstrated in mice, there is evidence in humans that testosterone synthesis during foetal life imply a coordinated action of foetal Sertoli cells and foetal Leydig cells (30). Therefore, a Sertoli cell dysfunction during foetal life may impair testosterone production by the foetal testis and lead to a lack of virilisation of the external genitalia at birth.

Furthermore, progressive degradation of testicular function with age was suggested in the literature based on several physical and hormonal observations. First, AMH was secreted during *in utero* life, but its levels were low at birth and in the neonatal period as discussed above (23–25), indicating gonadal dysgenesis. Herein, AMH levels were already low at birth. Then, a decrease in TV can occur during or after puberty (24); it was not significant herein perhaps because TV was initially too low. Finally, a progressive increase in the FSH and LH levels and a progressive decrease in testosterone and inhibin B levels with age were reported (9, 24, 25, 31). This pattern was observed herein mainly for FSH and LH levels but not inhibin B levels since the first value (at 12 years 7 months) was already too low. At 16 years 10 months, testosterone level was normal, but further degradation may not be excluded.

As expected given the low TV and low AMH and inhibin B levels, no sperm cells were retrieved in semen samples. Azoospermia was previously reported in *NR5A1* mutated patients (7), but varicocele could aggravate the spermiological phenotype in this case (32). Spermatogenesis could be improved 3 to 6 months after varicocele treatment (32).

Interestingly, some sperm cells were collected in the semen of some 46,XY mutated *NR5A1* patients (7, 8), and certain patients even fathered children naturally (25, 33–35). Among the latter, one patient had two children even though he carried an *NR5A1* pathogenic variant in a mosaic state in DNA extracted from blood leukocytes (25). One had two children at 30 and 33 years old but refused further investigations (35). One fathered five children before the age of 32 years and presented with increased FSH levels and undetectable AMH and inhibin B levels at 57 years old. However, no hormonal data were available when he was 32, and no sperm data were available for him or his boys (33). From the perspective of progressive hormonal function alteration, some authors suggested a progressive degradation of spermatogenesis with age that allows paternity in young men before spermatogenesis collapses (7, 23, 25, 31). However, this hypothesis remains to be confirmed by longitudinal sperm counts in *NR5A1* mutated 46,XY patients in whom spermatogenesis is preserved. Consequently, men carrying *NR5A1* pathogenic variants should be addressed for fertility preservation as early as possible after puberty; if mature sperm cells are retrieved, cryopreservation can thus be performed to ensure a timely medically assisted reproduction. A TESE was proposed to the patient herein when he was 17 years 10 months. Although it was performed sufficiently long enough after varicocele treatment to allow for the potential restoration of spermatogenesis, only one sperm cell was retrieved, thus preventing cryopreservation and intracytoplasmic sperm injection (ICSI) to be performed. In the literature (**Table 1**), one team failed to retrieve sperm cells in an 18-year-old man using TESE (9), while another retrieved sperm cells in three men (20, 31, and 35 years old) using micro-TESE after 3 months of vitamin E and clomiphene citrate for two of them (10). This discrepancy in TESE outcomes could be explained by different situations. First, based on the hypothesis of progressive spermatogenesis degradation, the age when TESE was performed may have impacted the outcomes. However, TESE retrieved sperm cells in the three older patients but failed in the youngest. Second, the TESE procedure performed: micro-TESE did not show better results for retrieving sperm cells than conventional TESE in men with non-obstructive azoospermia in a recent meta-analysis (36). Third, the wide spectrum of the disease without a clear phenotype–genotype relation likely impacts TESE outcomes. The fact that the same *NR5A1* pathogenic variant can cause different phenotypes in patients belonging to the same family (9, 33, 35, 37, 38) may suggest a possible polygenic inheritance or the intervention of additional epigenetic or environmental factors in the phenotype severity. In the case of polygenic inheritance, whole genome sequencing would be of great interest to find another mutated gene and to understand the spectrum of *NR5A1*-related diseases. Finally, features correlated with spermatogenesis function (39–41) like hormonal markers (FSH, LH, testosterone, and inhibin B), TV, and history of cryptorchidism are likely to affect TESE outcomes here: one patient in whom TESE retrieved sperm cells had a normal hormonal profile and normal TV. Unfortunately, we did not have access to the clinical description at birth or the follow-up of inhibin B levels since adolescence in all patients who underwent TESE to be able to suggest a relationship between the severity of the DSD and the TESE outcomes (9, 10).

The hormonal anomalies, sperm sampling, and TESE outcomes herein were consistent with the results of the pathological analysis of the biopsied testicular fragments. The pathological aspect observed herein was also consistent with the wide spectrum of testicular biopsy descriptions found elsewhere in 46,XY *NR5A1* mutated adults (8, 23, 42). The possible degradation of testicular function in terms of hormonal and sperm parameters with aging discussed above might parallel a progressive degradation of testis structure observable on the testicular biopsy, as suggested by Camats et al. (21). Nevertheless, if testicular biopsy finds germ cells and functional seminiferous tubules, future techniques of fertility preservation, such as the emerging *in vitro* spermatogenesis technology (43, 44), would be of great interest in *NR5A1* mutated patients with azoospermia.

Although overweight in *NR5A1* mutated patients has already been described (20, 45, 46), this feature was not found in all patients (46). Interestingly, the homozygous deletion of *NR5A1* in an SF-1 knock-out mouse model induced obesity (47). In line with this finding, some authors suggested the intervention of SF-1 in the development of the ventromedial hypothalamic nucleus, a central player in appetite regulation in humans (20, 46).

Finally, the presence of tetralogy of Fallot is surprising and was not reported elsewhere in association with a mutated SF-1. However, we could not exclude an additional genetic anomaly in other genes associated with DSD and/or tetralogy of Fallot, *GATA4* and *ZFPM2/FOG2* genes, for example (48, 49).

## 4 Conclusion

We report a case with a new *NR5A1* pathogenic variant addressed for fertility care. The physical, hormonal, and histological description of the testis could be integrated into the wide spectrum of 46,XY DSD related to mutated *NR5A1*. The patient presented with azoospermia since the first semen analysis when he was 16 years 4 months. A conventional TESE was performed at 17 years 10 months, but this procedure did not retrieve sufficient sperm cells for cryopreservation to perform an ICSI for future parenthood. These data extend the knowledge regarding fertility in *NR5A1* mutated patients. Further investigations in *NR5A1* mutated patients would help define a fertility care protocol in order to increase their chances of fertility.

## Data availability statement

The raw data supporting the conclusions of this article will be made available by the authors, without undue reservation.

## Ethics statement

The studies involving human participants were reviewed and approved by Ethics committee of Lyon University Hospital. Written informed consent to participate in this study was provided by the participants’ legal guardian/next of kin. Written informed consent was obtained from the participant/patient(s) for the publication of this case report.

## Author contributions

JT wrote the manuscript. IP, DM, FD, and FR-B supervised the laboratory procedures. JT, DM, LR, FD, FR-B, and IP interpreted the data. Patient care was performed by CG, EL, PB, SGD’E, BC, and IP. All authors contributed to the article and approved the submitted version.
